# The development of health literacy in patients with a long-term health condition: the health literacy pathway model

**DOI:** 10.1186/1471-2458-12-130

**Published:** 2012-02-14

**Authors:** Michelle Edwards, Fiona Wood, Myfanwy Davies, Adrian Edwards

**Affiliations:** 1 Department of Primary Care and Public Health, School of Medicine, Cardiff University, Heath Park, Heath, Cardiff CF14 4XN, UK; 2 School of Social Science, Bangor Univeristy, Neuadd Ogwen, LL57 2DG, UK

## Abstract

**Background:**

Inadequate health literacy has been associated with poor management of long-term health conditions and has been identified as a key social determinant of health outcomes. However, little is understood about how health literacy might develop over time or the processes by which people may become more health literate. Our objectives were to describe how patients with a long-term condition practice health literacy in the management of their health and communication with health professionals, how they become more health literate over time and their experience of using health services. We also sought to identify and describe the motivations, facilitators and barriers in the practice of health literacy in healthcare consultations.

**Methods:**

We designed a longitudinal qualitative study using serial interviews with 18 participants to explore their experiences of learning to manage their condition and their experiences of health literacy when participating in healthcare processes. Participants were recruited from patient education programmes and were interviewed three times over a period of 9 months. A framework approach was used to analyse data.

**Results:**

A model is presented that illustrates the development of health literacy along a trajectory that includes the development of knowledge, health literacy skills and practices, health literacy actions, abilities in seeking options and informed and shared decision making opportunities. Motivations and barriers to developing and practising health literacy skills partly reflected participants' characteristics but were also influenced by health professionals. Some participants developed their health literacy to a point where they became more involved in healthcare processes (including informed and shared decision-making).

**Conclusions:**

Patients with a long-term condition can develop health literacy skills over time and put their skills into practice in becoming more active in healthcare consultations. Our findings have implications for developing health literacy interventions aimed at patient involvement in healthcare processes and improved self-management of long-term conditions.

## Background

### Descriptions and measurements

Little is understood about how health literacy might develop over time or the processes by which people may become more health literate. Since the concept of health literacy was first introduced by Simonds [1], it has evolved from a basic description of the ability to perform health related tasks that require reading and computational skills [2], to cover cognitive capacities related to obtaining, processing, and understanding health information, leading up to decision making [3]. Nutbeam [4] extends the definition beyond a cognitive explanation and focuses also on social skills that he suggests are essential for interaction with others and society (e.g. skills in communication, negotiation and organisation). Nutbeam's focus on motivation presents health literacy as an action oriented concept rather than simply an intellectual capacity. Tones [5] has criticised newer definitions of health literacy for incorporating existing theoretical formulations of concepts such as social interaction and community empowerment. However, despite this the concept of health literacy has continued to evolve to reflect the contexts in which health literacy is considered to be important. For example, Zarcadoolas et al. [6] consider the outcomes of health literacy which are to "*make informed choices, reduce health risks and improve quality of life*". Zarcadoolas et al. [6] and Kwan et al. [7] have described health literacy as a 'generative' concept that develops over a lifetime. Nutbeam [8] describes two models of health literacy: the risk model emphasises the importance of communication and health service organisation that is tailored to the needs of low literate individuals, and the asset model where health literacy is described as an asset to be developed, and seen as an outcome of health education and communication.

Despite changes in the way health literacy has been conceptualised, the current body of health literacy research has been mostly based on cross-sectional studies using measurements informed by earlier definitions of health literacy as a cognitive capacity. The most commonly used measures used to investigate the relationship between health literacy and health outcomes are the Test of Functional Health Literacy in Adults [TOFHLA] [9], which focuses on reading comprehension, and the Rapid Estimate of Adult Learning in Medicine [REALM] [10], which focuses on reading ability.

Health literacy measures have been shortened for quicker use. For example, S-TOFHLA was later developed to include four numeracy items and two prose passages [11]. A shorter version of the REALM (REALM-R) is also available [12]. Extended measures also exist, for example, the Health Activities Literacy Scale (HALS) [13](developed in the US by the Educational Testing Service) has been specifically designed to assess activities that are not necessarily confined to traditional healthcare settings such as doctors' surgeries, hospitals and clinics, but those that take place in the home, at work or in the community. The HALS measures activities associated with the following:

• Health promotion (activities that enhance and maintain health)

• Health protection (understanding materials produced to attempt to safeguard health)

• Disease prevention (behaviours taken to prevent illness/detect disease)

• Health care and maintenance (activities to learn more about an illness or follow a prescribed regimen)

• System navigation (ability to read/understand bureaucratic and regulatory information *i.e. rights and responsibilities, informed consent*).

The HALS does take in more health contexts than other measures, but with 191 questions taking up to an hour to complete it may be too time consuming to use in most research. Hence, TOHFLA and the REALM (and their shortened versions) are the currently the most frequently used measures.

Health literacy measurements have helped identify a relationship between poor health literacy and adverse health outcomes. Lower health literacy has been associated with poor self-management [14,15], limited involvement in health care consultations and decision making processes [16,17], more emergency department use [18] and more hospital admissions [19,20].

### Health literacy and empowerment

The effective use of health information is critical to 'empowerment' [21]. Patients with limited health literacy may have limited knowledge and understanding of health that reduces their autonomy in self-care and decision making [22]. Because of limited understanding of what they are reading or what is being communicated to them in consultations people may also become disempowered, especially in consultations where healthcare professionals might be more paternalistic [22]. In our meta- study we found that health literacy mediated information exchange supported shared decision- making [23]. Communication styles of healthcare practitioners either facilitated information exchange and enabled empowerment or sometimes acted as a barrier to information exchange and disempowered patients. As people with better health literacy may be more empowered and also have better health outcomes, we expect that improvements in health literacy over time should lead to better self-management, better health outcomes (e.g., less hospitalisation [19]), more active involvement in health decision making and greater abilities to manage health conditions.

### Conceptualising health literacy from the patient perspective

Most descriptions of health literacy have come from the health promotion field [4,6,7]. However, few studies have attempted to conceptualise health literacy using qualitative methods. A recent model of health literacy has been developed based on qualitative research [24]. Interviewing participants and obtaining their perspectives, Jordan et al. [24] set out seven health literacy abilities related to seeking, understanding and using health information within the healthcare setting. These abilities are: knowing when and where to seek information, verbal communication skills needed to describe one's health issues and understand health professionals' responses, assertiveness (linked to successful communication), literacy, retaining and processing information, and skills in applying information.

Jordan's model considers these abilities in the context of broader healthcare system factors and can help inform healthcare professionals about patients' health literacy abilities and the personal barriers that may influence whether these abilities can be developed and put into practice. However, the model is based on single interviews with participants, so may not explain any changes or developments in health literacy abilities that occur over time and in different health contexts. Research is required into how patients may develop and adapt their health literacy skills and over time, how they put these skills into practice and what can be achieved in terms of access to health care and communication with health professionals.

### Aims

In this paper we consider different contexts where health literacy is important and the generative aspect of health literacy, using the definition of Kwan et al. [7]--*"People's ability to find, understand, appraise and communicate information to engage with the demands of different health contexts to promote health across the life-course"--*as a framework. We look at health literacy as an 'asset' rather than a 'risk' Nutbeam [8]. Thus, our focus was not on inadequate health literacy but the development of health literacy over time for people of all health literacy abilities and through self-learning and patient education. Our aim was to answer the questions:

• How do patients become health literate for their condition and how do they experience healthcare communication (including information exchange and informed/shared decision-making)?

• How does health literacy affect patients' experiences of using healthcare services in various contexts, and what are the facilitators and barriers to the development and use of health literacy skills?

We present the Health Literacy Pathway Model to describe how health literacy develops along a trajectory that enables individuals, supported by others, to seek, engage with and act on health information to manage their health and become more actively involved in healthcare consultations, in the context of various long term conditions. Facilitators and barriers to progression along the pathway are identified.

## Methods

### Study design and sample

We recruited 18 participants (aged 22-76) each with a long-term health condition. The sample was purposive in order to obtain the views and experiences of people with a range of conditions. Four participants attended a nurse-led structured diabetes education programme (X-PERT Diabetes Programme), five attended a nurse-led cardiac rehabilitation programme, five attended a generic lay-led self-management programme (the Expert Patient Programme), and four did not attend any programme (see Table 1 for participant details).

**Table 1:** Participant Characteristics

Participant code	Gender	Age	Condition(s)	Time living with condition	Level of education	Profession
**1CR**	Female	76	Heart surgery	Short-term	High school	Retired cashier for a pharmacy group

**2CR**	Male	77	Heart surgery/diabetes	Short-term	High School	Retired insurance broker

**3CR**	Female	73	Heart Surgery	Short-term	A Level	Hospital receptionist

**4CR**	Female	23	Rare and complex heart condition	Short-term	University	Physiotherapist

**5CR**	Male	54	Heart surgery	Short-term	University	Draughtsman

**1XP**	Male	72	Diabetes	Long-term	High school	Retired vending machine engineer/part time gardener

**2XP**	Female	53	Diabetes on insulin	Long-term	High school	Housewife

**3XP**	Female	52	Diabetes	Short-term	High school	Learning support worker

**4XP**	Female	69	Diabetes controlled by diet	Short-term	College	Part time secretary/retired bank worker

**1C**	Male	60	Heart surgery	Short-term	University	Part time Teacher

**2C**	Female	42	Mental illness	Long-term	High School/Military	Not in employment (previous army career)

**3C**	Male	40+	Back Pain	Short-term	High School	Not in employment (previously a cleaner)

**4C**	Female	45	Asthma	Long-term	University	Social Services Manager

**1EP**	Female	50	Epilepsy and Osteoarthritis	Long-term	College	Not in employment (previously a cook)

**2 EP**	Male	46	Bipolar disorder	Long-term	College/University	Construction

**3EP**	Female	49	Daughter with complex chronic illness	Long-term	College	Asylum seeker/was teacher

**4EP**	Female	66	Bipolar disorder	Long-term	University	Retired Dietician/Nutritionist - lecturer

**5EP**	Female	31	ADEM	Short-term	Some University	Ex healthcare assistant/social care worker

Participants in patient education groups were recruited with the assistance of the programme co-ordinators. Participants in the comparison group were recruited from a community education centre, one person was a tutor and the remaining three were undertaking evening classes. Potential participants were invited to take part in a study about their understanding of health information and how they use information in managing their health and communicating in consultations. Information packs containing a patient information sheet and consent form were distributed to potential participants and they were asked to return the consent form and their contact details to the researcher if they wished to participate. All participants who consented to be interviewed were informed that they would remain anonymous in the reporting of the study.

The Research Ethics Committee for Wales granted ethical approval for the study (reference 08/MRE09/54).

### Data collection

A longitudinal qualitative approach was used because of its utility in exploring evolving and complex processes, and to help develop the ongoing relationship with a participant that is necessary to explore sensitive topics [25]. Serial semi-structured interviews explored the development of health literacy and identified changes in attitudes, knowledge, and experiences over time. All participants were interviewed in their own home from January 2009-October 2009 by ME. Participants in the patient education groups were interviewed at three time points in order to capture changes in their understanding of their condition, self management skills, health care utilisation and health communication experiences over time (at the start of their programme, 2 weeks after completing the programme and approximately 12 weeks later). Participants in the comparison group were interviewed twice (initial time point and 20 weeks later).

Although, a general set of questions were asked to all participants in all patient groups, the interviews sometimes included specific questions for their condition, e.g. specific to cardiac conditions or diabetes (see Additional file 1 for examples of the initial interview guides). Participants were told that the research was about how they sought health information, their understanding of it, what they did with information and how they communicated with health professionals. The interview questions were more structured to begin with and evolved as new topics of interest arose within and across the groups of participants.

### Data analysis

The interview transcripts were analysed using a framework approach [26]. The framework takes into consideration pre-identified issues that the researcher wishes to investigate, but allows flexibility for new themes [26]. The framework approach was appropriate to explore whether participants had practised and developed health literacy according to existing definitions and models of health literacy, and discover new ways of describing health literacy in different health contexts. We analysed data within participants as well, i.e. across their two or three interviews, for evidence of how their health literacy may have developed over time. Thus, aspects of health literacy as described by Nutbeam, Zarcadoolas et al., Kwan et al. and others [4,6,7] were incorporated into the thematic framework. The five stages of analysis used in the framework approach were: familiarisation, identifying a thematic framework, indexing, charting and mapping and interpretation. The construction of the thematic framework and other stages of analyses were agreed by all authors.

### The framework process

*Familiarisation*--gaining an overview of the literature, research objectives and data (including proposal, literature review, interview topic guides, sample characteristics, interview and observation and themes found in the interview transcripts. (Themes were coded using the NVivo8 qualitative software programme) All the coding was completed by ME and FW and MD double coded 40 per cent of the data for reliability.

*Identifying a thematic framework*--this was constructed from the codes developed in the familiarisation stage.

*Indexing*--systematic coding of all interview transcripts using the thematic framework. Indexing can be done manually or by using a qualitative software package to code sections of data against the thematic framework. Indexing was carried out using NVivo8 software.

*Charting*--creating a set of thematic charts for each theme using a matrix format. Within each matrix, each participant is assigned a row and each sub theme is allocated a separate column. The data were charted using Microsoft Excel spreadsheets.

*Mapping and interpretation*--reviewing the charted data and analytical notes, comparing and contrasting participants' accounts, identifying patterns and connections in the data and seeking explanations for these within the data. These tasks helped define new concepts and create a set of typologies.

## Results

Based on familiarisation with the data and prior engagement with the literature on health literacy eight overarching themes were identified (see Table 2).

**Table 2:** Themes, sub themes and categories

Themes	Sub themes	Categories
1. Health knowledge	Knowledge of health in general and own health concerns	Knowledge of science and health
		Knowledge of condition
		Knowledge of health service
		Knowledge of patients' rights

2. Self management skills	Managing medication	Organising medications and managing a medication regime (self-injecting, taking pills)
	Self-monitoring	Self monitoring blood sugar/coagulation
	Managing a diet	Managing diabetes with diet

3. Active information seeking and use	Engaging with written materials	Reading medical reference books, dictionaries, leaflets, newspaper reports
	Accessing online information	Health-related websites, health organisations
	Using social media	Posting messages on discussion boards, web chat with other patients, using video sharing websites to view procedures
	Engaging with research	Reading research papers
	Critical appraisal of information and considering it within context	Assessing the reliability and quality of information and the source of information, assessing relevance of the information in context of own concerns

4. Actively communicating with health professionals	Preparation	Keeping a record of symptoms, preparing questions to ask in consultations
	Exchanging information	Bringing information to a consultation, discussing results, medications
	Expressing needs and concerns	Asking to change a medication, talking about problems, communicating preferences, asking for a referral to another service, asking for monitoring devices, asking to see results
	Conveying information	Reiterating health information given by one health professional to another
	Managing communication	Managing communication with multiple health professionals

5. Seeking and negotiating treatment options	Seeking treatment options	Seeking alternative treatment options online
	Negotiating medication or treatment	Asking doctor to try a new medication or alternative treatment method

6. Decision making	Desire for involvement	Making informed decisions about treatment preferences
	Opportunities for involvement	Taking part in shared decision making

7. Influences on health literacy	Negative influences (personal and professional barriers)	**Patients: **poor acceptance, compliance, reliance on health professionals for information, emotional barriers (shock fear, anxiety), avoidance of information
		**Health professionals: **poor communication styles, conflicting information
	Positive influences (personal and professional motivators, facilitators)	**Patients: **manage emotions (reducing fear), make sense of symptoms
		**Friends and family: **distributed health literacy skills
		**Health professionals: **GP support information seeking, pharmacy support with understanding of medications, nurse support with self-management, access to services and mediate communications with doctors

8. Health literacy outcomes	Develop knowledge, skills, understanding and coping	
	Active involvement in consultations	

These themes, sub themes and categories were used as a framework to analyse the data and define new concepts, create typologies, find associations, provide explanations and develop strategies for potentially defining and measuring health literacy.

### The development and practice of health literacy

Participants talked about their understanding of their condition, how they managed it and how they engaged with health information and health services. We used our thematic framework to help interpret their health literacy skills based on their accounts. We also identified areas where participants put their health literacy into practice. Participants sometimes progressed from developing their health knowledge to becoming active communicators and decision makers in their health care. Below are some of the abilities they demonstrated:

• Knowledgeable about their condition, health services and their rights as a patient;

• Skilled and organised in self-managing it;

• Actively involved in information seeking and use;

• Communicative with health professionals in an assertive manner;

• Able to seek and negotiate treatment options.

We suggest that health literacy was generative and as participants built on these abilities they became more health literate in managing their condition, in accessing and engaging with information and services, in discussions with health professionals and in negotiating and accessing treatments. However, there was variation in these abilities across our group participants, some had good knowledge and self management skills but were less involved in information seeking and less communicative in consultations.

#### Knowledge

Participants had developed some knowledge about their condition and continued to develop more knowledge over the time of the study. Most participants had a basic knowledge about health. However, two participants (5EP, 4CR) had worked in health settings and had a good knowledge of patients' rights and responsibilities and understood the role of health professionals. Some (4EP, 5EP, 5CR) had knowledge of science and biological processes associated with their condition and could understand their symptoms and how medications worked in their body.

Below is an example of the knowledge that one participant demonstrated about her recently diagnosed condition:

*"I fell ill in July 17^th ^last year with flu like symptoms and it developed into what they call um part of the encephalitis group but it's called ADEM (Acute Disseminated Encephalomyelitis). Initially it was called IDEM, it's called acute dissemination, it's to do with acute deterioration of your myelin sheath basically and with all this being attached to your nervous system is all being attacked." *(Participant 5EP, interview one)

Below participant 5EP demonstrates her knowledge of her rights as a patient and how the health service must provide access to her information.

"I know that because of the Freedom of Information Act you are entitled to see your notes and they charge you quite a lot of money if you want photocopies of them £50 um I know you are entitled to that and you are entitled to change your... and you are entitled to have a second opinion and without having prejudice about making that decision um and you are entitled to see your... well you should be entitled to see your patient care plan as well which is very important I think, so you know what's happening, um and that I kind of know from working in the hospital really."

#### Self- management skills

Participants were better able to understand how to manage their condition if they had good knowledge about their condition and good understanding of how medications worked in their body. Patients with diabetes particularly talked about managing their diet and medications to try to control their blood sugar. Participant 2XP had had diabetes for a long time but was gaining further information in the X-Pert Diabetes Programme. She had gained a better understanding of how her medication worked and how to manage her diet. Below she talks about her diabetes self management skills (managing medication):

*"I have an injection in the morning and it contains fast acting for your breakfast and then the slow acting will work on your lunch and then fast acting in the evening meal and then the slow acting for the rest. Then through the night again and I felt sometimes my biggest meal was lunchtime rather than dinner time. The time when I didn't have the injection and the slow was working. The slow was working lunchtime and I wanted the fast working so I felt that would bring my blood sugars down much lower and the only way you can do that is have an injection with every meal. That way I thought right I control what I'm eating, what I'm injecting and my activity what I'm doing, and work it all out accordingly, and that's basically what I'm doing"*. (Participant 2XP, interview two)

Some participants used self monitoring devices to monitor their blood and manage their condition. For example, participant 4CR had a good knowledge of how to manage her blood coagulation and was able to change from having her INR monitored in a clinic to self-monitoring at home. Both participant 1XP and 2XP also monitored their blood sugar several times daily.

#### Information seeking and use

In the first set of interviews participants talked about how they sought and critically engaged with various forms of health information, thus, using 'functional' and 'interactive' health literacy skills [4] to develop health knowledge and improve health literacy skills needed to manage their condition.

Participants engaged with written materials:

*"I've got a really good medical dictionary and I also find some of the sites, they underline something they kind of explain. I think it's... I'm not sure if it's patient... Or is it net doctor maybe that's quite good. I've come across quite a few of them that were explained or I just get my dictionary out and have a look"*. (Participant 5EP, interview 1)

Some accessed online health information:

*"I got more from going on to the MS society website because they seem to have a lot of information and it's quite good information and even though I went onto the Encephalitis website there still wasn't very much because MDEM was more common in children after inoculations, for some reason it can be a bacterial or viral disease so I just found I actually got more information by going onto the internet and going on to the MS site than I did from the hospital and going to the consultant." *(Participant 4EP, interview 2)

Some critically evaluated information sources:

*"If it's the companies themselves I'm slightly more sceptical than if it's sort of research done by universities about... you know a study of it and some of the findings and things, I feel a bit more reassured by that. Whether that's right or wrong I don't know but it seems a bit more objective so hopefully it's a bit more reliable but I do take the pharmaceutical trials and studies with a bit more um scepticism." *(Participant 4C, interview 1)

Some used social media (web chat and video sharing):

*"Having the GUCH online and being able to say... honestly being able to just put a question out there and say "when I breathe in why does it sound like my chest is popping"? I watched a few surgeries on You Tube as well in kind of... not in a morbid sense but I needed to see what happened." *(Participant 4CR, interview 1)

Some engaged with research and assessed relevance to them:

*"Yes, there's a really exciting one (drug trial) at the moment. I don't know if I'm meant to know, but I do know that my cardiologist is running it, so next time I see him I'm going to tackle him about it. It's a very exciting new drug that replaces Warfarin. So at the moment I'm doing 6.5 mg, the next day I've got to take 7 depending on what I eat. At the moment it tends to be quite steady but my INR still fluctuates, my level, whereas on this drug I would take one pill a day and it does the same as long as my INR is fine." *(Participant 4CR, interview 1)

#### Communication with health professionals

Participants talked about how they communicated with health professionals and discussed topics based on their understanding and more recently acquired knowledge of their condition. Some participants were motivated and able to seek and engage with new information, reflect on the information and then incorporate it into their consultations with health professionals. In some cases participants have had to communicate with multiple health professionals, sometimes having to assimilate information from various different specialist and sometimes conveying information between health professionals.

Some participants brought information to be discussed in a consultation:

*"I actually got the information off the internet and took it into him that kind of thing. He then went onto the website and got more information for himself"*. (Participant 5CR, interview 1)

Some participants expressed concerns about medications:

*"I approached the diabetes nurse and said I don't like the way things are going I feel like I'm injecting water because it doesn't seem to be doing anything." *(Participant 2XP, interview 1)

Some participants prepared questions (leading to negotiation of treatment):

*"I always prepare questions before I go. I have a notebook I use for medical consultations and I prepare information for him about my mental health and about my physical health and to discuss with him the medication that I'm on and whether it can be reduced." *(Participant 4EP, interview 2)

Some participants conveyed information between health professionals:

*"There is not always that communication straight away between my consultant and the GP they take a month for the letter to get to the GP and to say what needs to be done whereas if I see him and I tell him what's coming he kind of knows, and if he's disagreeing then he's got a chance in the meantime to actually post a phone call and say... which has happened in the past when there's been a disagreement over monitoring my certain drugs, um and I've gone back and told him and straight away... He's dealt with it where if he had waited for a letter it would have delayed things even more so there has been a lot of bouncing of information." *(Participant 5EP, interview 2)

Some participants managed information from multiple health professionals:

*"I'm managing 5 different consultants between Cardiff and London. I'm the only one that gets information from all of them I mean they all talk to each other but they don't retain the information because they have got so many patients so I find it easy to manage myself and just ask a GP when I need something but my rheumatology team are really good and they are the ones I go to for advice." *(Participant 4CR, interview 3)

#### Active involvement in consultations

Participants who were informed, motivated and confident through developing their knowledge and health literacy skills became more actively involved in their care and were motivated to communicate their needs and concerns to health professionals. Thus they were able to make informed decisions about their treatment preferences.

One participant was able to draw on her knowledge of her diabetes treatment to negotiate a new treatment regime with her diabetes nurse:

*"I said "I'm not happy with the way things are going and I want to try something else" and I said "I want to try four injections a day I said instead of having two mixed in the morning have a mixed insulin and two at night I want three fast acting and slow for the night and try a completely different."" *(Participant 2XP, interview 2)

Some were able to seek out (sometimes with the help of others) alternative treatments or new treatment and negotiate them with their health professionals:

*"I did some research and I found a liquid aspartame that I can buy from Boots and she went OK. She had a look at it and discovered it only gives you a normal dose of iron and I need like loads so she said well there is another liquid form, it's like a juice, and I'm still taking that about three months later and that's had no side effects"*. (Participant 4CR, interview 2)

*"I did think about going on a recent drug um called Byetta, it's quite new and I said could you have a look on the internet to see what they say about it and um I could perhaps ask my doctor then whether it would be suitable for me which is what we did yesterday"*. (Participant 2XP, interview 2)

*"Yes there's this new therapy called DBT which means dialectical behaviour therapy, it's quite new and it's supposed to have good results and I have spoken to my doctor about it and she feels that it's a good way to go and she's trying to arrange that for me*." (Participant 2 C, interview 2)

Where the opportunity arose, some participants were able to take part in shared decision making:

*"We discussed the use of statins because my cholesterol was a little bit high um and um we talked about it jointly really and he said he wasn't... he said he could put me put me on statins but he asked me what I felt about it basically and at that stage I said no um but only after discussing it not just a straight no"*. (Participant 5CR, interview 2)

*"When I first went on to beta-blockers for high blood pressure I kind of discussed it with the doctor and you know we decided... I took his advice basically but I was aware that there was a variety of different medications"*. (Participant 5CR, interview 2)

### Influences on the development of knowledge and health literacy skills

There were three broad factors that influenced whether participants had the motivation and opportunity to become knowledgeable and health literate, and whether they could become actively involved in consultations. These were personal motivations, emotional factors and access to facilitators.

#### Personal motivations

Some were motivated to seek and engage with information to help explain the way they were feeling and to understand symptoms and effects of medication. For example, some participants sought information from internet sources to understand why they might be experiencing symptoms such as problems with vision (4CR), dizziness (5EP) and skin rashes (5CR).

#### Managing emotions

A range of emotional factors motivated participants' engagement with information and their potential to develop their knowledge and health literacy skills. Participant 5EP developed her health literacy by seeking information about her condition and potential difficulties that may arise in the future; partly limiting her emotional reaction to the effects should they occur.

*I'm doing a lot of research at the moment into um what my condition is and what to look out for... I think it's very important to at least know or have an idea of what could possibly happen otherwise if you don't you are just sitting in a... your oblivious world and then when it does happen it can hit you twice as hard*. (Participant 5EP, interview 1)

After surgery to replace a heart valve, participant 4CR became anxious about some of the potential effects of surgery. She engaged with media to view television documentaries and You Tube clips of the same surgical procedures that had been performed on her. The understanding she had gained through her information seeking helped her make sense of some of the effects of by-pass surgery that she was experiencing, and reduce her anxieties about potential effects. However, it took some time for her to feel comfortable to engage with such information. The data extract below was from interview two (6 months after surgery).

*I had to see everything that had happened so I can understand. All of these silly little things go through your mind and then until you actually see how they stitch the valves in place, and they just caught, and it goes into place, and how they tug it around to make sure it's secure, and how they test it afterwards. Only then I was like ok I'll chill out now I know that it's safe and it's secure and I'm not going to leak blood everywhere*. (Participant 4CR, interview 2)

#### Friends and family

Friends and family acted as *health literacy mediators*, sharing their knowledge and health literacy skills to access information, interpret and analyse that information, communicate with health professionals on behalf of, and in collaboration with participants. They supported participants in their health literacy actions by seeking and using information to consider treatment options and influenced their decision making.

Some received support from family or friends when communicating with health professionals, helping participants to build their knowledge and understanding and become more active in consultations. The nature, complexity, amount or timing of the information was sometimes difficult to engage with, when participants felt stressed or anxious about their diagnosis. Participant 5EP took a friend with a health service background, to draw on her knowledge and critical skills, to more actively participate in her consultations.

*"I always have someone with me, even from working in the health profession I was always taught if you are going to see a doctor, have someone with you because you don't always process what's been said. I've got a friend who has an NHS background who sits there with me because there are certain things she's questioning as well..." *(Participant 5EP, interview 1)

#### Health professionals as facilitators

Some health professionals were supportive to the development of health literacy, although others created barriers that prevented or discouraged participants from developing and practising their health literacy skills. Supportive health professionals facilitated the development of health literacy by encouraging participants to engage with information before making a treatment choice. For example, participant 2XP was informed of the use of patches for pain relief by her GP, and asked if she would consider them as a convenient treatment option. He then suggested she look at the information about patches that was available on the internet, which she did before deciding.

*He said well go home and have a think about it, have a look on the internet. He said you will be able to find all the information you want have a read about it. He said, and you come back in a month and then you know if you want to try it*. (Participant 2XP, interview 2)

Participant 5EP perceived her GP to be very interested in learning more about her condition and assisted her in seeking information. Participant 4 C had a similar experience with her GP in managing her asthma and allergy condition. Their GPs helped facilitate the development of health literacy through encouraging an exchange of information, support in seeking information and guidance on what to do with that information in terms of self-management decisions.

Pharmacists also provided participants with information about their medications and supported them in managing their medication regimes. Consultations and discussions with pharmacists facilitated the development of health knowledge and health literacy skills and provided participants with information they could then discuss further with their GP.

*I had the pharmacist at the hospital phone me to double check I was having blood tests regularly and ask if everything had been explained to me and they double checked*. (Participant 5EP, interview 1)

Nurses acted as health literacy facilitators by assisting information seeking, supporting transitions to new medication regimes and facilitating access to further information and services. Overall, nurses helped facilitate development of health literacy by helping participants increase their medication knowledge, teaching self-management skills, facilitating access to other services and communication between participants and doctors, and introducing treatment options.

*She visits me every week to make sure I'm ok, to make sure I'm taking my meds. You know keep me up to date, and then sort of be the go-between between me and my um consultant as well*. (Participant 2 C, interview 1)

### Barriers to the development of health literacy and using health literacy skills

Several barriers inhibited the development and use of health literacy skills and limited opportunities for participants to become actively involved in healthcare consultations. These barriers are broadly categorised as personal barriers, emotional barriers and professional barriers.

#### Personal barriers

Personal barriers such as a lack of personal motivation, not accepting a diagnosis and a tendency to be compliant to medical advice prevented participants from carrying out health actions such as accessing health care services, and limited active involvement in healthcare consultations. Personal barriers tended to be based on attitudes towards health and help seeking behaviour.

Participant 4XP had a problem accepting her diabetes; this affected her development of health literacy in a number of ways: she did not want to disclose her diabetes to other people, even to friends and work colleagues who were diabetic themselves. This prevented her extending her knowledge of diabetes and self-management skills through learning from others.

Some participants relied on health professionals as their only source of health information, engaging with little or no other health information. Although some were happy to comply with medical instructions, it limited their understanding of their medication and limited progression towards active involvement in healthcare consultations.

*"I'm afraid I'm one of these people, I'm told to take tablets and I take them and that's it but I notice with other people they were asking questions and they really must read those leaflets that are inside the tablets inside the box that are very detailed... I don't. I'm given the tablet and I take them"*. (Participant 1CR, interview 1)

#### Emotional barriers

Unpleasant emotional reactions (e.g. shock, fear and anxiety) prevented some participants from engaging with or processing information. For example, participant 1CR was temporarily shocked about needing by-pass surgery and her anxiety prevented her from processing the initial information that her consultant gave her about her condition. Participant 4XP had unpleasant memories of family members' experiences of diabetes and found it difficult to accept her diagnosis of diabetes. Her embarrassment along with her fears and anxieties associated with diabetes deterred her from accessing support from her GP.

*"I don't really want to go to the doctors. I've got to go I know, but I'm afraid of what they are going to say like you know. That it's gone worse or have this or... I'm sort of putting it off all the time like you know." *(Participant 4XP, interview 3)

Emotional reactions to information were changeable over time and some participants were ready to engage with more information as they became better able to cope with it. Participant 4CR exemplified this: at first the seriousness of her condition and anxiety about the possible prognosis made her stop seeking and engaging with information, but over time she had overcome her anxiety, and was able to seek and engage with information to develop an understanding of her condition, manage it, effectively communicate with a number of health professionals and make self-management and treatment decisions in collaboration with health professionals involved in her care.

#### Health professional barriers

Health professionals' poor communication skills undermined the opportunity for participants to make use of their health literacy skills in consultations and created a barrier to exchanging information. Some referred to 'not being given enough information' and 'not being listened to'. Others felt that information that they brought with them or their ideas about treatment were dismissed by their health professional. Information was sometimes withheld based on incorrect assumptions concerning participants' information needs and their ability to understand health information. For example, participant 3XP wanted to see her blood test results but her GP would not let her have them.

*"I've had terrible trouble getting out of him what my results are from the consultant at the hospital and um you know my blood results. His attitude was well you don't know what they mean. And I say yes, but I do know what they mean and if I don't I will find out." *(Participant 3XP, interview 2)

There were also instances of conflicting information from different health professionals which can be confusing for patients who must try to evaluate which advice to accept. For example, participant 4CR had received different instructions about managing her Warfarin dosage from her primary and secondary care team. Although her trust in the medical profession led her initially to comply with the advice, she also then fully developed her own understanding of how her INR should be managed.

### Synthesis: the health literacy pathway model

**Figure 1:**
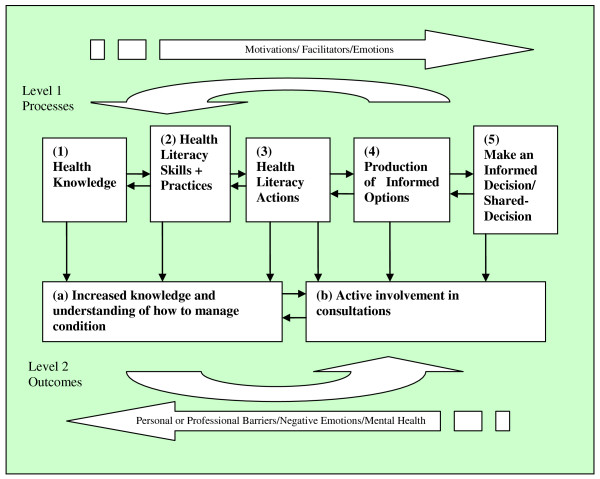
**The Health Literacy Pathway Model**.

Our longitudinal qualitative analysis of how participants' developed and used their health literacy skills to become more active and empowered patients enabled us to map a set of stages that participants progressed through as they increased their knowledge and understanding of their condition, learned how to manage it, actively participate in discussions with health professionals, and make informed self-management and treatment decisions. Progression through these stages is presented in a theoretical model mapping a *health literacy pathway *from health knowledge towards decision-making (see Figure 1). The model includes five stages along a pathway; each stage requires a more complex set of health literacy abilities. As participants progress through each stage, they develop their health literacy further and gain the opportunity to feel more empowered. Health literacy *processes *are represented in the five stages of the Pathway Model and health literacy *outcomes *are represented as running parallel to those stages.

#### Stage one: building health knowledge

This stage represents a person's basic knowledge about health in general and knowledge about their own health concerns. This knowledge is formed through reading about health, interactions with health professionals and/or health educators, discussions with friends and family and interactions with media health information. Some participants had a basic general knowledge of health issues but little knowledge of a newly diagnosed condition; others had been living with a condition for some time and had built up a substantial amount of knowledge.

#### Stage two: developing health literacy skills and practices

The health literacy skills stage represents competencies in listening, speaking, arithmetic, problem solving, and decision making that are used in information seeking and use of information, (e.g. using computer to seek information, skills in information seeking, critical analysis of information), and self-management skills such as understanding medication (e.g. anticoagulant dosages) and blood sugar measurements. Health literacy practices are the *tasks *that participants carried out with these skills (e.g. searching for health information, balancing diet with medication, self-monitoring blood sugar). Health literacy skills at this stage can increase the development of health knowledge, thus there is a feedback relationship between these stages. One example of this feedback relationship is the ability to successfully access and use information in order to further one's knowledge of a condition.

#### Stage three: displaying health literacy actions

Health literacy actions represent active involvement in one's health and relates to communication with health professionals where someone is asking for a treatment or service, expressing concerns and desires for information and services, requesting and negotiating medications and contributing to consultations in terms of information exchange.

#### Stage four: the production of informed options

Potential treatment options were sometimes presented in healthcare consultations by health professionals. However, some participants were able to produce their own list of potential treatment options after engaging with health information sources and informal discussions with friends and family. Skills used at stage three of the pathway model are helpful to producing options (stage 4) and making a more independent informed decision (stage 5).

#### Stage five: making an informed decision

At this stage options have been produced, deliberated and firm decisions are made about treatments or self-management tasks. Participants who reached this stage have moved along the Pathway Model from stage one to stage five. However, their transition may not have been linear; with some movement between the stages in both directions.

### Transition along the health literacy pathway towards decision making

Participants became more knowledgeable about their condition through self-learning or through the patient education programmes in which they were participating, and used their knowledge and skills to become active in consultations and make decisions about their health. For example, after learning about the existence of self-monitoring devices for INR (stage 1) participant 4 CR carried out research (stage 2) and thought about ways to obtain a device (stage 3), considered her options (stage 4) to acquire funding for a device to help her manage her warfarin medication, she then entered discussions with health care providers and charities to negotiate funding for the device and the strips to use with it (stage 3). After some negotiation with her primary care service she made a decision (stage 5) to buy the device herself and obtained funding for the strips from her GP surgery (stage 3). Thus, her decision is a result of acquired knowledge, skills in accessing and acting on information, consideration of available funding options and negotiations with health professional and charity organisations.

*I went in to ask for funding for my self-tester and they didn't have the funding, they referred me to a charity that could help and I got in touch with the charity and the charity were going to fund me but they didn't have money either. So they were trying to be helpful but at the end of the day I bought my own tester but the doctor has said that she can prescribe the test strips on prescription so that's taken away the burden of £150 every six months or so*. (Participant 4CR)

Participant 4CR continued to self-monitor her INR level and self-manage her warfarin medication throughout the time of the study.

### Health literacy outcomes

Some participants developed their knowledge and skills in order to gain a better understanding of their condition, extend their skills in managing their condition and develop ways of coping with it. Others became more actively involved in their care, to use health information to identify options for treatment and care, and enter active discussions with health professionals. The outcomes of these two stages for health literacy were categorised as: a) increased knowledge and understanding of how to manage and cope with the condition and b) active involvement in consultations. Below are examples of health literacy outcomes:

#### A) Increased knowledge and understanding of how to manage and cope with condition

Participant 1XP was quite knowledgeable about diabetes and was good at self-managing it. He engaged with lots of written information about diabetes provided in the media and provided by Diabetes UK. He monitored his blood sugar several times a day and kept a record of it, he felt he had good control over his blood sugar and was adherent to the medication regimes that were prescribed by his GP. He tended to be more compliant and was not particularly active in healthcare consultations.

#### Both A) Increased knowledge and understanding of how to manage and cope with condition and B) Active involvement in consultations

Participant 4CR actively sought information online and learned about her condition from internet sources such as charities and support groups in addition to her interactions with multiple health professionals. She built up an understanding of her condition and how to manage it over time and was very active in seeking alternative medications and self-monitoring materials that she required. She managed a series of appointments with a number of health professionals and specialists in her condition.

Participant 2XP also appeared to be competent in these two outcomes. She had developed an understanding of diabetes through interactions with health professionals, membership of Diabetes UK and engagement with information that her husband would look up online for her. She had skills in self-managing her condition but also strived to manage it better and sought alternative medications that might help her bring her blood sugars down further. She actively engaged in consultations with health professionals.

## Discussion

### Principal findings

Our study advances a broader understanding of health literacy by showing health literacy to be a multi dimensional construct that develops over time, across different health contexts and through social interactions. Our study shows health literacy in action and how it develops as a result of increasing knowledge from engaging with both written and human information sources (i.e. lay sources, educational sources and professional sources). In the Pathway Model, health literacy develops along a trajectory towards a number of milestones that include greater knowledge, improved self management and participation in (informed and shared) decision making. One important feature of the model is that it highlights health literacy as both a process and as an outcome.

Parallel to the process and outcome of becoming more health literate is the concept of empowerment. We found that participants who followed the pathway through the stages perceived themselves to be more empowered through their greater understanding of their condition and confidence and ability in communicating with health professionals. However, the important role of health care practitioners was also evident in that they can empower people, facilitating health literacy, or disempower, limiting health literacy.

### Strengths

We used longitudinal qualitative methods to generate a contextualised view of health literacy and a model of how health literacy may develop over time. Using the framework approach to analyse data [26] enabled us to consider the number of ways health literacy has been described so far and incorporate those into our exploration of how health literacy was practised and developed in our sample of participants. The diversity of long-term conditions that were featured in this study helped demonstrate a number of different health contexts in which health literacy is important. For example, including newly diagnosed diabetes patients and those who had the condition for some time enabled the exploration of how long-term sufferers had developed their health literacy and how more recently diagnosed participants had to quickly learn how to manage diabetes. Including patients who had previously experienced a heart event or undergone major heart surgery helped identify how health literacy is drawn upon when reflecting on past information and health experiences in order to move forward. Some participants had more serious conditions where the diagnosis was still being considered, multiple healthcare professionals were involved, several treatment methods were being tried out or prognosis was unclear (e.g. participants 4CR, 4EP and 5EP). We had a range of participants from different educational and occupational backgrounds in our study. Just over half of participants (10) had developed their condition in the short-term (last 1-3 months) and the remainder had developed their condition and had been self managing over a longer term (more than 1 year). Some of our participants did not work as they were unable to because of their condition, two participants spoke English as a second language, half of our participants had attained college or university level education and half had high school level education. We acknowledge that some participants worked in health-related fields and some had a good level of health knowledge and a base-line of health literacy skills that may have enabled them to improve their health literacy more quickly than others. However, two of our most health literate participants had recently developed very rare conditions with complex self-management needs for which they had no previous knowledge. Our study differs from other studies of health literacy that have focussed on low-literacy populations. We were able to explore the concept of health literacy as an asset [8] and show how health literacy might develop in people of all educational abilities and across different occupations.

### Limitations

One criticism that could be put forward is that the longitudinal element covered an average period of only 20 weeks. There were sufficient data to detect some initial reported behaviour changes, new health literacy practices, social developments (i.e. changes in communication styles) and developments in learning, but longer follow up period would have been useful to explore whether such developments were sustained and how health literacy may have developed further, especially for those who were more recently diagnosed or had more complex conditions. Our sample is not representative of a low-literacy population and may have included participants with better literacy skills because they had chosen to take part in patient education programmes. However, we aimed to show how health literacy might develop within a range of contexts, including situations where participants were more educated and had a good baseline of health knowledge and literacy skills.

### Implications for policy and practice

A number of policy initiatives, for example in the UK [27,28] have been put forward to address patient education and the quality of and access to health information. Patients need support to develop and maintain their health literacy skills over their lifespan. The implementation of structured diabetes education (X-PERT) and self-management programmes (EPP) have been part of these initiatives. The conceptual ideas produced by this study may help provide a framework by which to evaluate their effectiveness in developing health literacy and recommend necessary changes.

There is also an ongoing policy focus on patient choice about treatment and partnership with health care providers [29]. Adequate health literacy is essential for patient involvement in their health care [30] and is crucial for patients to make optimal choices. Health care policy makers and providers need to be aware of health literacy orientated strategies to encourage and support patients to make such choices.

Professional barriers to health literacy identified in this study can be addressed through strategies to educate professionals about the development of health literacy and the range of tasks that require and comprise adequate health literacy. By recognising the different dimensions and stages of health literacy, health professionals may be able to communicate health information in a way that is tailored to best develop patients' understanding of their long-term condition and how to manage it. Furthermore, healthcare professionals may be able to encourage patient involvement in other healthcare processes (e.g. information exchange and informed/shared decision-making when coming to new conditions or scenarios).

### Future research

Our research adds to a growing body of research development in health literacy [31]. Some of the health literacy related concepts in our findings seem to be similar to some concepts used to inform an evaluation tool to assess the impact of health education programmes for people with a long-term condition (e.g. the Health Education Impact Questionnaire- HeiQ) [32]. However, Nutbeam [7] suggests that different health literacy measurement tools may be required for different stages and ages in order to account for the different social contexts in which health literacy is relevant. Our Pathway Model can inform the development of measures that can be used at different stages in health, for different conditions, at different stages of health literacy development and their evaluation over short-term periods and over the life course. Health literacy concepts developed here could be incorporated into the design of patient education programmes aimed at developing health literacy, improving self-management and communication skills. Appropriate baseline and post intervention health literacy measures based on our Pathway Model could be used in studies to establish whether health literacy has developed through such educational interventions. However, we acknowledge that our hypothesized Health Literacy Pathway Model needs to be tested and confirmed before incorporation into the development of such interventions or evaluations.

## Conclusions

This study has shown that becoming health literate is an ongoing process that develops over time through a range of health experiences and encounters within different health contexts. A focus on health literacy across different health contexts is helpful to understand how the development of health literacy plays a part in health outcomes. In a recovery or rehabilitation context, health literacy helps some reflect, understand, cope with and recover from health events that had occurred in the past. For others who may have recently developed a long-term condition, health literacy helps them cope and develop self- management skills, and for those who may be uncertain about their future health developing health literacy helps provides foresight of future risks and preparation and adjustment of life plans.

One important goal for policy makers, patient educators and health professionals should be to develop, implement and evaluate new strategies and interventions to develop health literacy in all patients with a long-term condition. Efforts to test and promote such interventions would need appropriate health literacy measures that incorporate the dimensions of health literacy and capture changes in health literacy over time. Our findings on how health literacy was practised and developed by participants in our study may be useful to inform the development of such health literacy interventions at the individual level (within consultations), and through the delivery of group-based patient education and health promotion programmes.

Future efforts to improve health literacy for all groups of patients with a long-term condition could help raise health literacy at a public level and enable patients with a long-term health condition to be autonomous and empowered decision makers in their health. In turn, this could contribute to reducing health inequalities. Bringing patients together to develop health literacy at the community level could enable the delivery of structured health education/information and make use of available social capital that helps distribute health literacy through groups of patients.

## Competing interests

The authors declare that they have no competing interests.

## Authors' contributions

ME carried out data collection and analysis and drafted the manuscript, FW, MD and AE supervised the project and contributed to the analysis of data and draft of the manuscript. All authors read and approved the final manuscript.

## Pre-publication history

The pre-publication history for this paper can be accessed here:

http://www.biomedcentral.com/1471-2458/12/130/prepub

## Supplementary Material

Additional file 1 **Initial interview guides**. Click here for file
