# Supplementary material for: The development of health literacy in patients with a long-term health condition: the health literacy pathway model
Source: BMC Public Health. 2012 Feb 14;12:130. doi: 10.1186/1471-2458-12-130 (PMC3305618; doi:10.1186/1471-2458-12-130)
Supplement: Additional file 1 — Initial interview guides. [file 1471-2458-12-130-S1.DOC]

**Interview 1:**

**Research Question**

What is the meaning of health literacy to patients with a chronic condition, how does this compare to definitions of health literacy.

**Objective**

Describe the meaning and experience of health literacy skills for patients with a chronic condition and compare this meaning to functional/interactive/critical health literacy and other dimensions.

**Before we start, do you have any questions you would like to ask me?**

Can you tell me what long term health condition(s) you have?

How long have you had this condition?

What kind of things do you need to know about in order to live as comfortably as you can with [given condition]? *[Prompt participant to list what they need to know, explore each item further if necessary]*

How important is it to you to clearly understand all these things you need to know?

*[If they express importance ask why it is important, what does it mean for them?]*

Do you have any particular skills in managing your condition?

*[Give an example of a skill and prompt participant to list their skills, explore each skill further if necessary]*

How have you learned the knowledge and skills that you have?

*[Prompt participant to think about how they came to know how to manage their health]*

Are their any things that you don’t understand or any skills that you don’t have but would like to learn more about?

*[Prompt participant to list, explore item further if necessary]*

How would you prefer to learn these skills?

*[Prompt participant to think about ways that they prefer to learn, e.g. classes, from friends, from doctor, nurse etc]*

**Research Question**

What evidence is there of a range of patients health literacy practices- according to given definitions of health literacy

**Objective**

Describe how patients' understandings and abilities concerning their health and their health literacy practices compare with given definitions of health literacy

Do you look for information about your health condition?

What resources do you use?

*[Give examples, internet, magazines TV etc]*

How much do you understand the health information that you have sought about your health condition? *[What is understood and not understood? What are the reasons?]*

How do you work out which sources of information are reliable?

How often does the information usually apply to your circumstances?

Do you make decisions based on information you have sought about your health condition? *[What decisions?]*

Has any information that you have discovered led you to make an appointment to see your doctor to discuss further? *[Probe for examples]*

Do you know anything about patients’ rights and access to medical records? *[Have you ever accessed your medical records?]*

Do you usually read the information leaflet that comes with your medication? [*What do you pay attention to, mostly?]*

Do you pay attention to the dosage instructions and the possible risk of side effects?

Do you belong to any support groups or other groups that involve anyone with the same medical condition as you have? *[If yes, probe for details and ask about their experience and how it benefits them]*

**Research Questions**

What are patient’s experiences with using healthcare services across different health contexts in relation to their health literacy abilities?

What are the facilitators and barriers to exercising health literacy skills?

**Objective**

Describe how health literacy may affect patients' experiences of using healthcare services in various contexts and identify possible facilitators and barriers.

What healthcare services do you use? (e.g. GP visits, nurse visits, outpatient visits, out of hours service, physiotherapist)

How often do you come into contact with these services?

How would you describe your understanding of how to use or interact with these services? *[Do you know who to make contact with and how to reach them, do you keep regular appointments? Do you know what you need to do if you run out of medication or if there is an emergency? Do you know how to contact a doctor out of hours?]*

Do you ever talk to your doctor about any health information you have discovered? *[Probe further to ask what information]*

For example, have you ever brought information to a consultation to show it to the doctor or nurse? *[If no, ask why]*

*[If yes]*

What kind of response do you get?

How much new information do you learn from your consultations with various health professionals?

Do they give information to you in a way that you understand?

*[If no, why do you think this is?]*

Do you ask your doctor/nurse for more information about your health?  *[If yes, what kind of information?]*

*[If no, why not?]*

Do you ever need for your doctor or nurse to clarify any information or advice they have given you? *[If yes, what information is that?]*

Have you been involved in making decisions together with a doctor/nurse? *[Probe for examples]*

Do you feel that you have enough input into those decisions?

Have you ever had to sign a form to consent to a treatment or procedure? *[Probe for experiences]*

To what extent do you feel you have been able to understand the risks and benefits of the treatment or procedure before you have signed the form? *[What is understood and what is not understood? What are the reasons?]*

Is there anything else that you would like to discuss with me today about your understanding of health information or using healthcare services?

**Interview 2:**

**Research Question**

What are patients’ views on their experience of participating in a self management or patient education course and what they have gained from it?

**Objective**

Describe what patients achieve from their experience of participating in a self-management programme (keeping within a focus on health literacy).

How would you describe your experience of taking part in a patient education course?

What was the best part of the experience for you?

What new knowledge or skills did you learn from the course?

Who did you learn from?

What are the most useful skills you have learned from the course?

How much did you learn that you didn’t already know or understand?

Do you intend to make any changes to the way you manage your health based on any new knowledge or skills?

What are the changes that you would make?

What did you learn about communicating with healthcare professionals?

How do you think that what you have learned on the course will influence how you communicate with your doctor in the future?

Is there anything else that you would like to tell me about your experience of the course?

**Interview 3:**

**Research Question**

What are patients’ experiences of applying health literacy skills obtained through self-management education to their interactions with healthcare professionals and how does this affect information exchange and shared decision-making in healthcare consultations?

**Objective**

Describe the experience of information exchange and shared decision-making in healthcare consultations following participation in self-management programmes

How much communication have you had with healthcare practitioners since completing the course?

How would you describe your experience of communicating with healthcare practitioners since completing the course?

Have there been any changes in the way you communicate with healthcare practitioners?

How has your understanding of information given during your consultation been since you completed the course?

Have you had the opportunity to ask your GP, nurse or specialist further questions about any information you have learned during the course?

Have you talked to your GP, nurse or specialist about the course and what you have learned?

*[If, no why not?]*

*[If yes, what was their reaction?]*

Have you been involved in making any decisions with your healthcare practitioner?

Is there anything else you would like to tell me about your communication with your healthcare practitioner since you completed the course?
